# Loss of Effector and Anti-Inflammatory Natural Killer T Lymphocyte Function in Pathogenic Simian Immunodeficiency Virus Infection

**DOI:** 10.1371/journal.ppat.1002928

**Published:** 2012-09-20

**Authors:** Namita Rout, Justin Greene, Simon Yue, David O'Connor, R. Paul Johnson, James G. Else, Mark A. Exley, Amitinder Kaur

**Affiliations:** 1 New England Primate Research Center, Harvard Medical School, Southborough, Massachusetts, United States of America; 2 Wisconsin National Primate Research Center, University of Wisconsin-Madison, Madison, Wisconsin, United States of America; 3 Beth Israel Deaconess Medical Center, Harvard Medical School, Boston, Massachusetts, United States of America; 4 Yerkes National Primate Research Center, Emory University, Atlanta, Georgia, United States of America; NIH/NIAID, United States of America

## Abstract

Chronic immune activation is a key determinant of AIDS progression in HIV-infected humans and simian immunodeficiency virus (SIV)-infected macaques but is singularly absent in SIV-infected natural hosts. To investigate whether natural killer T (NKT) lymphocytes contribute to the differential modulation of immune activation in AIDS-susceptible and AIDS-resistant hosts, we compared NKT function in macaques and sooty mangabeys in the absence and presence of SIV infection. Cynomolgus macaques had significantly higher frequencies of circulating invariant NKT lymphocytes compared to both rhesus macaques and AIDS-resistant sooty mangabeys. Despite this difference, mangabey NKT lymphocytes were functionally distinct from both macaque species in their ability to secrete significantly more IFN-γ, IL-13, and IL-17 in response to CD1d/α-galactosylceramide stimulation. While NKT number and function remained intact in SIV-infected mangabeys, there was a profound reduction in NKT activation-induced, but not mitogen-induced, secretion of IFN-γ, IL-2, IL-10, and TGF-β in SIV-infected macaques. SIV-infected macaques also showed a selective decline in CD4^+^ NKT lymphocytes which correlated significantly with an increase in circulating activated memory CD4^+^ T lymphocytes. Macaques with lower pre-infection NKT frequencies showed a significantly greater CD4^+^ T lymphocyte decline post SIV infection. The disparate effect of SIV infection on NKT function in mangabeys and macaques could be a manifestation of their differential susceptibility to AIDS. Alternately, these data also raise the possibility that loss of anti-inflammatory NKT function promotes chronic immune activation in pathogenic SIV infection, while intact NKT function helps to protect natural hosts from developing immunodeficiency and aberrant immune activation.

## Author Summary

Several African nonhuman primate species such as sooty mangabeys are naturally infected with SIV and maintain high levels of viral replication without developing AIDS. SIV-infected natural hosts do not show evidence of increased chronic immune activation, a feature that distinguishes them from AIDS-susceptible SIV-infected Asian macaques. In this study we compared natural killer T (NKT) lymphocytes, a unique subset of innate T lymphocytes with anti-inflammatory properties, in AIDS-resistant and AIDS-susceptible hosts. Sooty mangabey NKT cells retained normal functionality following SIV infection and were more potent than macaque NKT cells in their ability to produce interferon-γ and secrete anti-inflammatory cytokines. In contrast, NKT cells of SIV-infected macaques were markedly hypo-functional with regards to secretion of anti-inflammatory and effector cytokines and showed an association between loss of CD4^+^ NKT cells and increased immune activation. These findings suggest that dysfunctional NKT cells may promote increased immune activation in AIDS-susceptible hosts while intact effector and anti-inflammatory NKT cells could help to prevent immunodeficiency and increased immune activation in natural hosts.

## Introduction

Absence of chronic immune activation is a key distinguishing feature that separates nonpathogenic simian immunodeficiency virus (SIV) infection in natural hosts from pathogenic lentiviral infection in HIV/SIV-infected humans and macaques [1]. Primary SIV infection studies have shown that natural hosts such as sooty mangabeys and african green monkeys develop increased immune activation early in SIV infection [2], [3], [4], [5]. However, unlike SIV-infected macaques, the increased activation is short-lived and rapidly declines to pre-SIV infection levels [6], [7], [8], [9]. The discrepancy in immune activation levels appears to be confined to non-virus-specific activation because the magnitude of SIV-specific cellular immunity during acute and chronic SIV infection is comparable in sooty mangabeys and rhesus macaques and thus, does not account for the difference in level of T cell activation between the two hosts [3], [4], [10]. An understanding of mechanisms by which acute immune activation is rapidly resolved and remains quiescent in natural but not non-natural hosts will provide insight into the basis of chronic immune activation in pathogenic lentiviral infection.

Natural Killer T (NKT) lymphocytes are unconventional T cells with immunoregulatory properties that belong to the innate immune system and recognize glycolipid antigens presented on the non-polymorphic MHC I-like CD1d molecule [11]. Classical NKT cells express an invariant TCRVα chain (Vα14-Jα18 in mice and Vα24-Jα18 in humans) paired to a restricted TCRVβ repertoire [11]. They also express several markers of the NK lineage, have cytolytic activity, and display an activated or memory phenotype. NKT lymphocytes do not require prior sensitization and rapidly secrete copious amounts of both Th1 and Th2 cytokines, including IL-2, IFN-γ and IL-4 upon antigen encounter. Consequently, they modulate activation of other immune subsets including dendritic cells, NK cells, and B and T lymphocytes, and influence both innate and adaptive immunity [11], [12], [13]. As a result of their immunomodulatory and effector abilities, NKT lymphocytes can influence diverse functions, including tumor surveillance, anti-microbial defenses, and maintenance of self-tolerance [14], [15].

Several studies have shown that NKT lymphocytes are affected by HIV/SIV infection in vitro and in vivo. NKT cells expressing CD4 and HIV co-receptor molecules show increased susceptibility to HIV infection in vitro [16]. They are rapidly depleted in vivo in HIV-infected humans and SIV-infected pig-tailed macaques and the depletion appears to be due to both direct infection of CD4-expressing NKT and Fas-mediated apoptosis of CD4-negative NKT lymphocytes [17], [18], [19]. HIV proteins including Nef and Vpu downregulate CD1d expression on antigen presenting cells suggesting that lentiviruses have evolved strategies to inhibit NKT as well as conventional MHC class I-restricted T cells in vivo [20], [21], [22]. However, the functional consequences of NKT loss in HIV infection remain unclear. In light of their immunoregulatory function and role in bridging innate and acquired immunity, it is likely that NKT loss could result in both enhanced immune activation and suppression of microbial immunity in HIV-infected humans. Comparative studies of AIDS-resistant natural hosts and AIDS-susceptible non-natural hosts of SIV infection may provide valuable insight into the role of NKT lymphocytes in AIDS pathogenesis.

We recently reported that sooty mangabey NKT lymphocytes are unique in lacking a CD4^+^ subset, a feature likely to prevent NKT depletion following SIV infection [23]. We hypothesized that the presence of SIV-resistant NKT lymphocytes may be responsible for suppression of chronic immune activation in SIV-infected sooty mangabeys. To investigate the role of NKT lymphocytes in down-modulating immune activation in SIV-infected natural and non-natural hosts, we compared the frequency and functionality of NKT cells in AIDS-resistant sooty mangabeys to that of AIDS-susceptible cynomolgus macaques and rhesus macaques. Our data show significant differences in the phenotype and function of sooty mangabey NKT lymphocytes compared to NKT lymphocytes in rhesus and cynomolgus macaques. CD4^+^ NKT lymphocytes were readily detected in both macaque species and were significantly decreased in SIV-infected animals. The surviving NKT lymphocytes in SIV-infected macaques showed global functional loss with hypo-proliferation, decreased production of effector and anti-inflammatory cytokines, and skewing of residual NKT function towards IL-6, a pro-inflammatory cytokine. We also observed a significant correlation between CD4^+^ NKT depletion and increased CD4^+^ memory T cell activation. Our results suggest that NKT depletion and dysfunction are factors contributing to increased immune activation in SIV-infected macaques. In contrast, preserved NKT lymphocyte function as observed in SIV-infected sooty mangabeys may be important for controlling immune activation and maintaining intact immune responses in nonpathogenic SIV infection.

## Results

### Distinctive CD4/CD8 phenotype of sooty mangabey and macaque NKT lymphocytes

**Figure 1:**
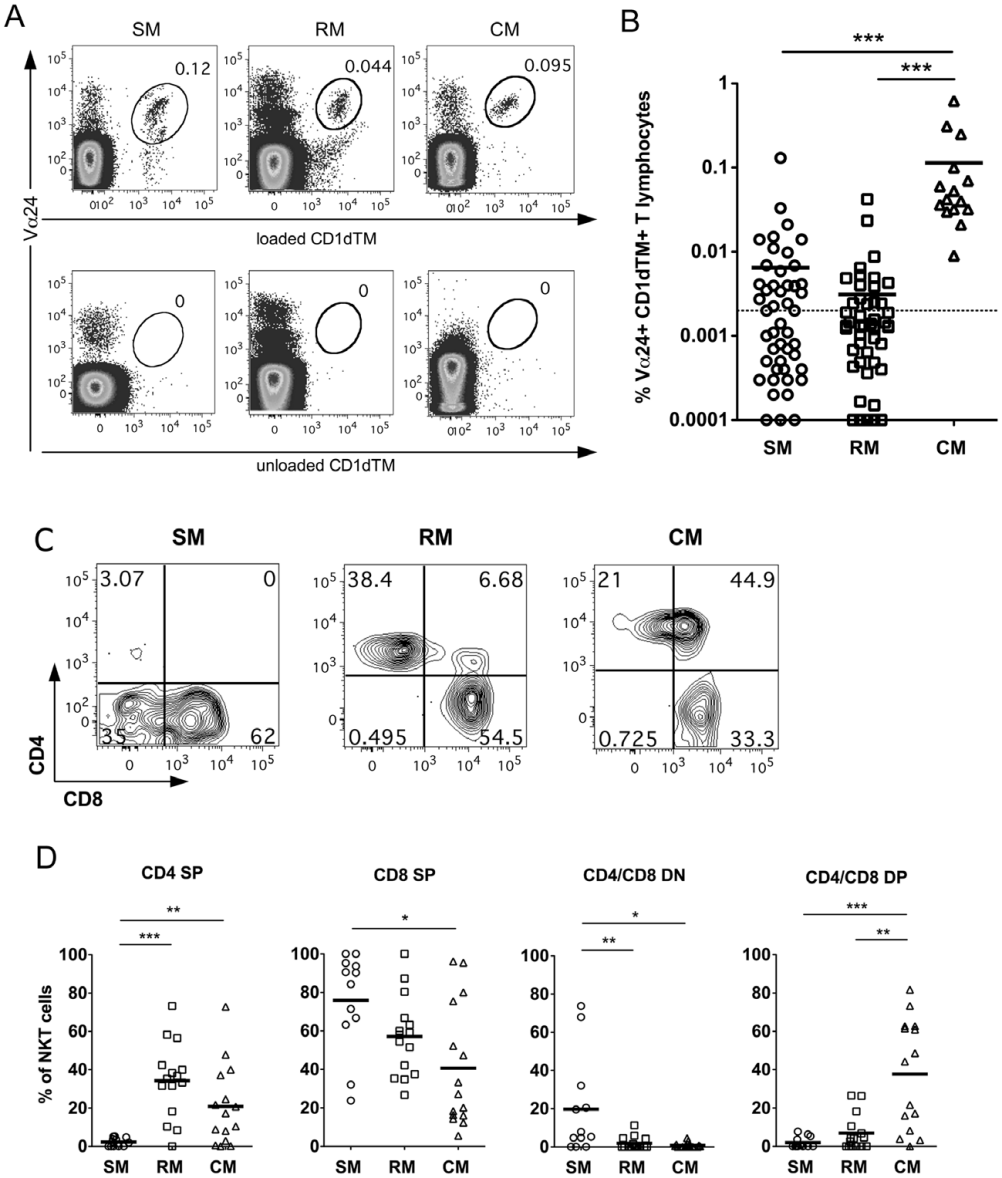
Comparison of NKT lymphocytes in AIDS-susceptible and AIDS-resistant nonhuman primate species. A) Dot-plots of *ex vivo* peripheral blood NKT lymphocytes in one SIV-negative sooty mangabey (SM), rhesus macaque (RM) and cynomolgus macaque (CM). Gated CD3^+^ T lymphocytes co-staining for Vα24 and CD1d tetramers loaded with PBS-57 were used to identify NKT lymphocytes (top panel). Co-staining for Vα24 and unloaded CD1d tetramers served as a negative control (bottom panel). B) Frequency of peripheral blood NKT lymphocytes in SIV-negative SM (n = 50), RM (n = 48) and CM (n = 15). Dotted line at 0.002% denotes the cut-off for the lower limit of flow cytometric detection of NKT lymphocytes. C) Representative contour plots of CD4 and CD8 surface expression on NKT lymphocytes in SM, RM and CM. D) Frequencies of NKT subsets with CD4^+^CD8^−^ (CD4 SP), CD8^+^CD4^−^ (CD8 SP), CD4^+^CD8^+^ (CD4/8 DP), and CD4^−^CD8^−^ (CD4/8 DN) phenotype in SIV-negative SM (n = 12), RM (n = 15), and CM (n = 15). * *P*<0.05, ** *P*<0.01, *** *P*<0.001, Mann-Whitney U test. Horizontal bars denote mean values.

We previously reported that NKT lymphocytes in sooty mangabeys are unique in lacking expression of the CD4 molecule [23]. To investigate differences between AIDS-resistant and AIDS-susceptible species, we performed a comparative cross-sectional analysis of NKT lymphocytes in SIV-negative sooty mangabeys (n = 50), Indian rhesus macaques (n = 48) and Mauritian cynomolgus macaques (n = 15). Invariant NKT (iNKT) lymphocytes, defined as Vα24-positive T lymphocytes binding to α-galactosylceramide (αGalCer) analog PBS-57-loaded CD1d tetramers, were detected in the peripheral blood of all three species (Fig. 1A). Owing to the rarity of circulating NKT lymphocytes, a minimum of 200,000 CD3^+^ T lymphocyte events were collected to ensure that detection of NKT frequencies <0.01% reached a power of ≥80% at P-value<0.05 [23]. Moreover, based on staining with unloaded CD1d tetramers, a cut-off value of 0.002% was used to define the lower limit of flow cytometric detection of NKT lymphocytes [23]. Ex vivo circulating iNKT lymphocytes with frequencies ≥0.002% were detected in 15 of 15 (100%) cynomolgus macaques, 15 of 48 (31%) rhesus macaques, and 24 of 50 (48%) sooty mangabeys (Fig. 1B). iNKT lymphocytes in cynomolgus macaques (mean = 0.1%, range = 0.008 to 0.6%) were present at a significantly higher frequency compared to rhesus macaques (mean = 0.003%, range = 0 to 0.04%) and sooty mangabeys (mean = 0.006%, range = 0 to 0.13%), and were in the range observed in humans [24].

Despite the inter-species difference in NKT frequency, both cynomolgus and rhesus macaques shared several phenotypic similarities and differed from sooty mangabey NKT lymphocytes with regards to the surface expression of CD4 and CD8 molecules (Fig. 1 C–D). The majority of peripheral blood NKT lymphocytes in sooty mangabeys were distributed between a CD8^+^ and CD4/CD8 double-negative (DN) phenotype and there was a paucity of CD4^+^ and CD4/CD8 double-positive (DP) NKT cells (Fig. 1C). In contrast, CD4^+^, CD8^+^, and DP NKT lymphocytes were present in both macaques species, but DN NKT lymphocytes were rarely seen (Fig. 1C). CD4^+^ NKT lymphocytes consisted predominantly of CD4 single positive T cells in rhesus macaques, but included both CD4 single positive and CD4/CD8 DP T cells in cynomolgus macaques (Fig. 1C–D). CD8^+^ NKT lymphocytes were detected in all three nonhuman primate species, with the highest mean frequency being present in sooty mangabeys (Fig. 1D).

### Functional differences in sooty mangabey and macaque NKT lymphocytes

The CD4/CD8 phenotype of NKT lymphocytes can be associated with distinct functional signatures. Human DN NKT lymphocytes have been associated with Th1 functionality/bias, while CD4^+^ NKT subsets can produce both Th1 and Th2 cytokines [25]. We have previously shown that the CD8^+^ and DN NKT subsets in sooty mangabeys have a Th1 and Th2 bias respectively [26]. To investigate functional differences in ex vivo NKT lymphocytes of SIV-negative mangabeys and macaques, PBMC were stimulated with the NKT ligand αGalCer presented on stable transfectants of CD1d-expressing C1R cells (CD1d/αGC) and cytokine secretion in supernatants was measured by ELISA as previously described [23].

**Figure 2:**
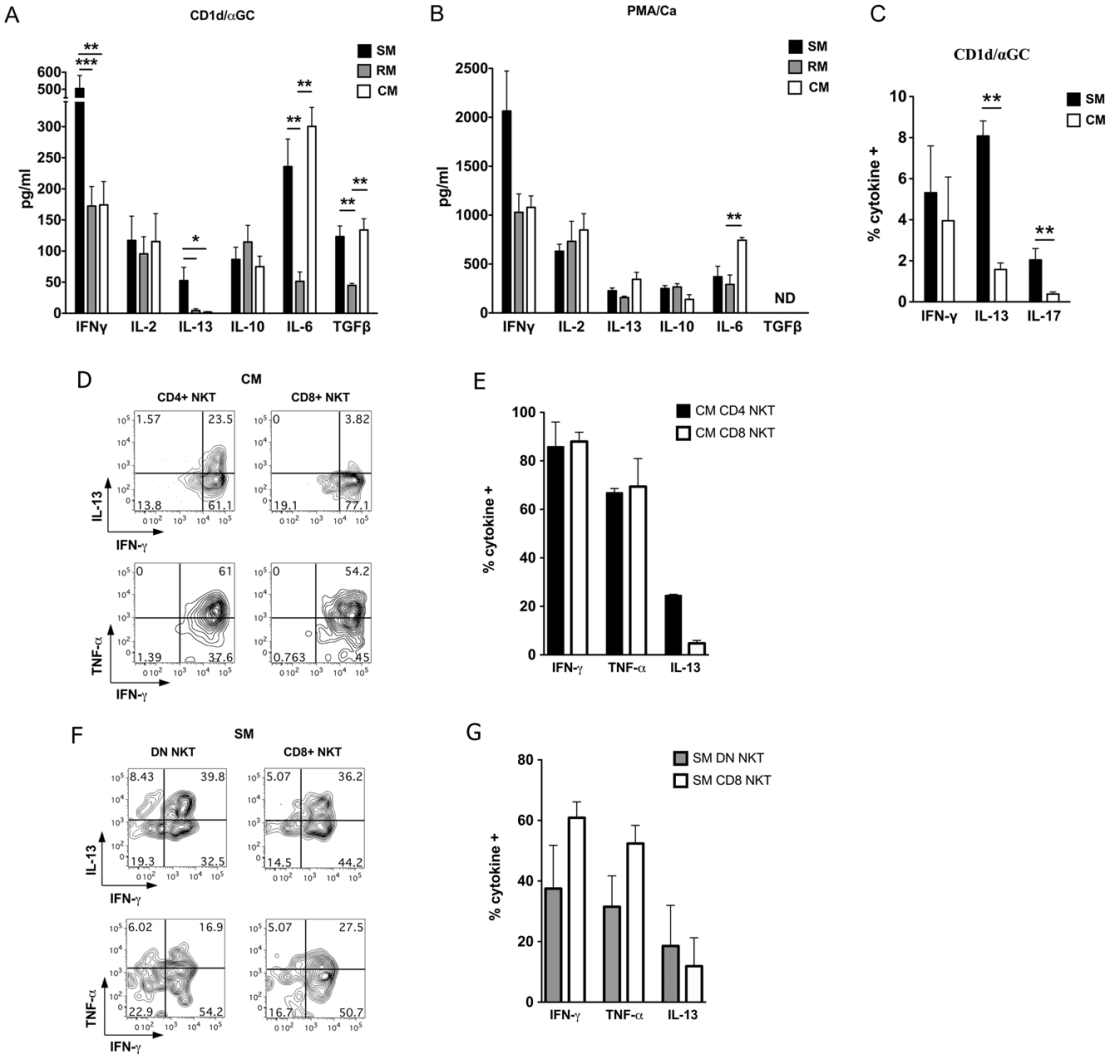
Th1 and Th2 cytokine secretion profiles of sooty mangabey (SM), rhesus macaque (RM) and cynomolgus macaque (CM) NKT lymphocytes. Cytokine ELISA for IFN-γ, IL-2, IL-13, IL-10, IL-6, and TGF-β with culture supernatants collected 24 h following *in vitro* stimulation of SM, RM, and CM PBMC with (A) NKT ligand α-GalCer presented on CD1d expressing C1R cells (CD1d/αGC) or (B) PMA/Ca ionomycin (PMA/Ca). C) Intracellular cytokine staining (ICS) data showing mean percentages of 12 h IFN-γ, IL-13, and IL-17 production by in vitro expanded SM and CM NKT lymphocytes (stimulated with CD1d/αGC). D) Contour plots showing ICS for IL-13, TNF-α, and IFN-γ in ex vivo CD4^+^ and CD8^+^ NKT lymphocyte subsets from one SIV-negative CM following 4 hour PMA-stimulation. E) Bar graphs showing mean percentages of cytokine-positive NKT cells in the CD4^+^ (filled bars) and CD8^+^ (open bars) CM NKT subsets from two SIV-negative CM. F) Contour plots showing ICS for IL-13, TNF-α, and IFN-γ in ex vivo DN and CD8^+^ NKT lymphocyte subsets from one SIV-negative SM following 4 hour PMA-stimulation. G) Bar graphs showing mean percentages of cytokine-positive NKT cells in the DN (filled bars) and CD8^+^ (open bars) SM NKT subsets in three animals. Data on gated CD3^+^CD1dTM^+^ shown. Error bars denote Standard Error of Mean. * *P*<0.05 and ** *P*<0.01 Mann-Whitney U test.

**Figure 3:**
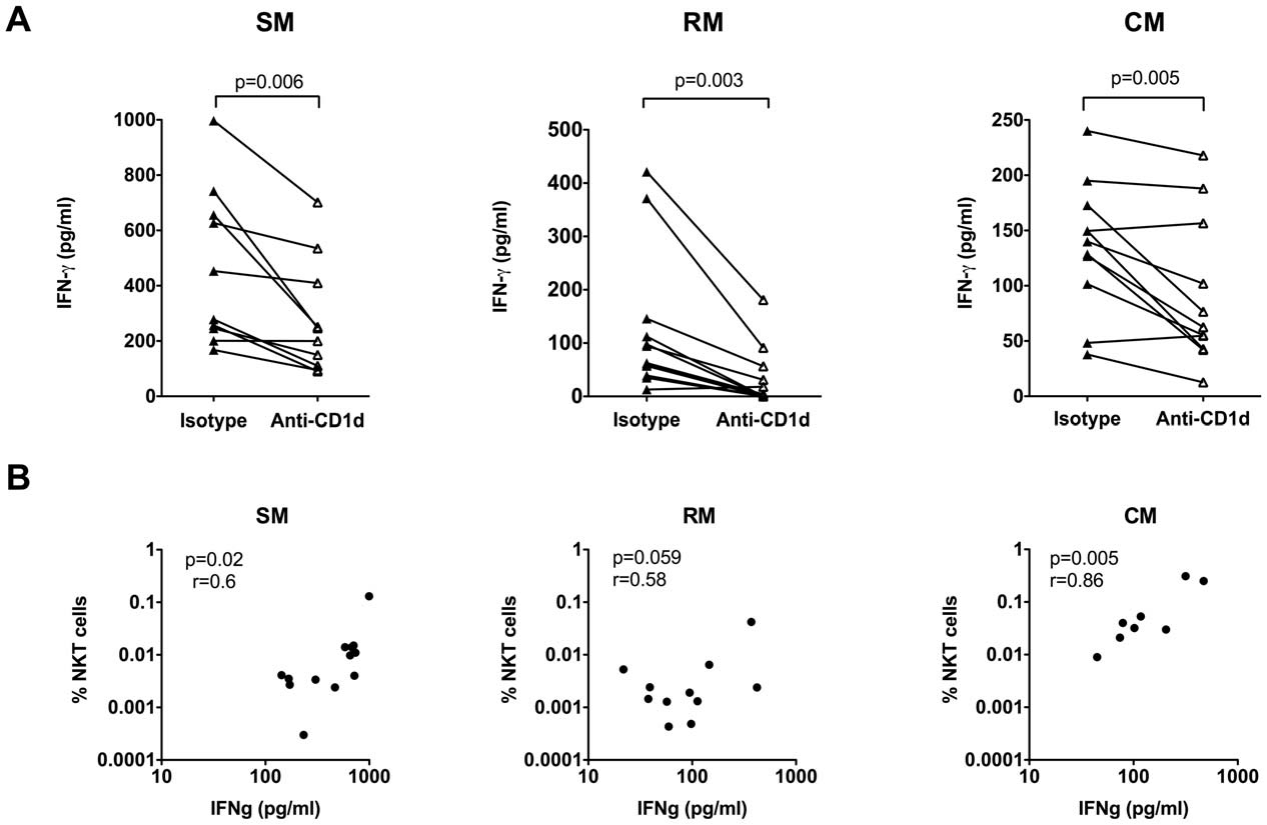
Cytokine production on CD1d/αGC stimulation is mediated by NKT lymphocytes. A) IFN-γ production by SM, RM and CM NKT lymphocytes following 24 hours *in vitro* stimulation with CD1d/αGC in the presence of anti-human CD1d blocking antibody or isotype control antibody as a negative control. *P* values shown for paired t test. B) Pearson's correlation test on ex vivo frequency of NKT lymphocytes and IFN-γ production in SM, RM, and CM lymphocytes following 24 hours *in vitro* stimulation with CD1d/αGC.

Ex vivo NKT lymphocyte stimulation in PBMC from SIV-negative animals of all three species induced the Th1 cytokines IFN-γ and IL-2, and the immunomodulatory cytokines IL-10, IL-6, and TGF-β upon overnight CD1d/αGC stimulation (Fig. 2A). The Th2 cytokine IL-13 was produced on NKT activation of mangabey lymphocytes but was absent or detected at very low levels on NKT activation of rhesus macaque and cynomolgus macaque lymphocytes (Fig. 2A). In addition to IL-13, sooty mangabey NKT lymphocytes also produced significantly higher levels of IFN-γ compared to macaque NKT lymphocytes (Fig. 2A). These differences were specific for NKT activation because lymphocytes from all three species produced comparable levels of IL-13 and IFN-γ on mitogen stimulation with PMA (Fig. 2B). The specificity of the CD1d/αGC-stimulated NKT activation response was confirmed by suppression of IFN-γ, IL-2, IL-13, and IL-10 production following addition of anti-CD1d antibody (Fig. 3A and data not shown).

Flow cytometric analysis of intracellular cytokine secretion in expanded NKT cells generated after in vitro stimulation of PBMC with CD1d/αGC for one week, confirmed a higher frequency of IL-13-producing NKT lymphocytes in sooty mangabeys compared to cynomolgus macaques (Fig. 2C). In vitro expanded NKT cells also revealed a small population of IL-17-secreting NKT in sooty mangabeys (Fig. 2C) that were not evident on stimulation of ex vivo NKT lymphocytes in peripheral blood (data not shown). These data suggested that sooty mangabey NKT were functionally distinct from Mauritian cynomolgus macaques with regards to their capacity for IL-13 and IL-17 secretion.

To investigate whether the CD4/CD8 NKT subsets in macaques were functionally different, we used flow cytometry to examine the Th1/Th2 cytokine profile of ex vivo CD4^+^ and CD8^+^ NKT lymphocytes in PBMC stimulated with PMA for four hours (Fig. 2D–E). Because of the low frequency of NKT lymphocytes in the peripheral blood of rhesus macaques, this analysis was only feasible in cynomolgus macaques. CD4^+^ and CD8^+^ NKT subsets of cynomolgus macaques showed no significant difference in their ability to produce the Th1 cytokines IFN-γ and TNF-α. However, the Th2 cytokine IL-13 was almost exclusively produced by CD4^+^ NKT lymphocytes (Fig. 2D–E). Similar analysis of PMA-stimulated PBMC in sooty mangabeys revealed that unlike cynomolgus macaques, IL-13 was produced by both CD8^+^ and DN NKT lymphocytes in sooty mangabeys (Fig. 2F–G). A trend for greater Th1 cytokine production by CD8^+^ NKT and greater Th2 functionality in DN NKT subsets of sooty mangabeys did not reach statistical significance (Fig. 2G). Overall, macaque CD4^+^ NKT lymphocytes appear to be similar to human CD4^+^ NKT lymphocytes and mangabey DN NKT lymphocytes with regards to increased Th2 functionality.

Given the differences in ex vivo NKT frequency between the three nonhuman primate species (Fig. 1B), it was surprising to observe that NKT cells in sooty mangabey PBMC produced significantly more IFN-γ compared to both cynomolgus and rhesus macaques (Fig. 2A). Even though the frequency of peripheral blood NKT lymphocytes in cynomolgus macaques was 10- to 100-fold higher than sooty mangabeys and rhesus macaques, there was no difference in the level of NKT-activation induced IFN-γ production in the two macaque species (Fig. 2A). Consistent with these findings, a significant positive correlation between the frequency of peripheral blood NKT lymphocytes and NKT-activation induced IFN-γ production was observed only within each species and did not apply across species (Fig. 3B and data not shown). Thus, the differences in frequency of NKT lymphocytes between species are not predictive of their functionality. These data highlight the discrepancy between NKT frequency and function in nonhuman primates and raise the possibility that mangabey NKT lymphocytes are more potent on a per cell basis compared to macaque NKT lymphocytes.

### Loss of CD4^+^ NKT lymphocytes in SIV-infected macaques

**Figure 4:**
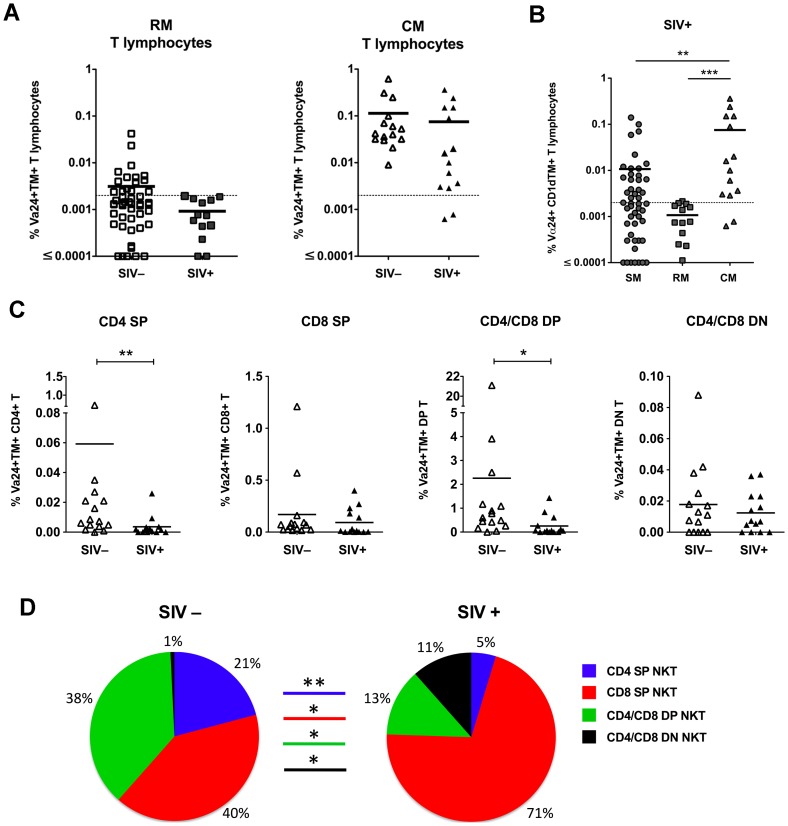
Frequency and subset distribution of NKT lymphocytes in SIV-infected macaques. A) Percentage of total NKT lymphocytes in peripheral blood T lymphocytes of SIV-negative and SIV-infected RM and CM. Dotted line at 0.002% denotes the limit of detection. B) Percentage of total NKT lymphocytes in peripheral blood of SIV-infected SM, RM, and CM. ** *P*<0.01, *** *P*<0.001 Mann-Whitney U test. C) Comparison of frequencies of CD4^+^ SP, CD4/CD8 DP, CD8^+^ SP and CD4/CD8 DN NKT subsets in peripheral blood of 15 SIV-negative CM and 14 SIV-infected CM. Horizontal bars denote mean values. D) Pie charts showing frequency of CD4^+^ SP, CD4/CD8 DP, CD8^+^ SP and CD4/CD8 DN NKT subsets in total NKT lymphocytes of SIV-negative and SIV-infected CM. * *P*<0.05 and ** *P*<0.01 Mann-Whitney U test.

We next compared the effect of chronic SIV infection on NKT lymphocytes in macaques and mangabeys. We previously showed that the frequency and subset distribution of NKT lymphocytes remains intact in naturally SIV-infected sooty mangabeys with chronic infection [23]. NKT frequencies in SIV-infected macaques were evaluated in a cross-sectional analysis of 13 rhesus macaques and 14 cynomolgus macaques infected with SIVmac239 for at least three months prior to analysis (Table 1). Similar to sooty mangabeys, the total frequency of peripheral blood NKT lymphocytes did not significantly differ between SIV-negative and SIV-infected cynomolgus macaques (Fig. 4A). Although the NKT frequency in rhesus macaques also did not significantly differ in the absence or presence of SIV infection, the small number of animals with NKT frequency above the limit of detection makes this conclusion less definitive (Fig. 4A). Consistent with these findings, the frequency of circulating NKT lymphocytes in SIV-infected mangabeys and macaques showed a similar hierarchy to that observed in SIV-negative animals, with the NKT frequency in cynomolgus macaques being significantly higher compared to rhesus macaques and sooty mangabeys (Fig. 4B).

**Table 1:** Status of SIV-infected rhesus macaques (RM) and cynomolgus macaques (CM) at the time of NKT analysis.

	Median (Range)*	
	SIV+ RM (n = 13)	SIV+ CM (n = 14)
Weeks post SIV challenge	78.5 (22–102)	45 (14–140)
Log_10_ plasma SIV RNA copies/ml	#5.5 (1.5–7.3)	5.16 (1.7–6.8)
Percent circulating CD4^+^ T lymphocytes	50.7 (27.7–69.3)	28.8 (4.3–53.3)

* At time of NKT analysis.

# Data available on six RM at time-point of NKT analysis.

The NKT subset analysis performed in SIV-infected cynomolgus macaques, showed a significant decline in the frequency of CD4^+^ SP and DP NKT lymphocytes in SIV-infected macaques (Fig. 4C). As a result of the preferential loss of CD4^+^ NKT lymphocytes, the proportion of CD8^+^ and DN NKT subsets within the total NKT lymphocyte population showed a significant increase in SIV-infected as compared to SIV-negative cynomolgus macaques (Fig. 4D). The low circulating NKT lymphocyte frequencies precluded reliable assessment of changes in CD4/CD8 NKT subset frequencies in SIV-infected rhesus macaques (data not shown). These data are consistent with reports of the loss of CD4^+^ NKT lymphocytes in HIV-infected humans and SIV-infected pigtail macaques [16], [17], [19].

### Attenuated NKT lymphocyte function in SIV-infected macaques but not sooty mangabeys

**Figure 5:**
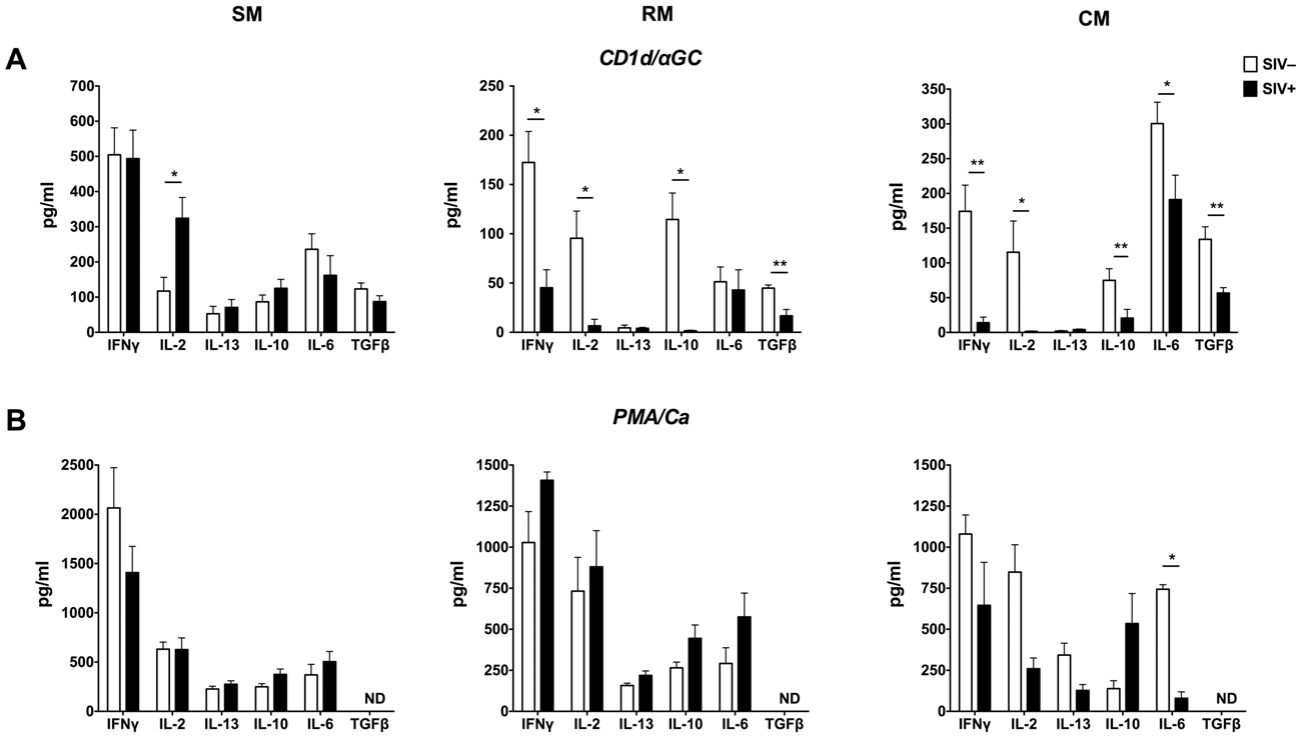
NKT cell hypo-function in SIV-infected macaques. Amounts of IFN-γ, IL-2, IL-13, IL-10, IL-6, and TGF-β production in culture supernatants collected 24 h following *in vitro* stimulation of PBMC from SIV-negative and SIV-infected SM, RM and CM with (A) CD1d/αGC and (B) PMA/Ca ionomycin. Open bars denote mean values of cytokines secreted from SIV-negative animals (n = ≥6) and filled bars denote mean values of SIV-infected animals (n = ≥4) from each species. Error bars denote SEM. * *P*<0.05, ** *P*<0.01 Mann-Whitney U test.

Ex vivo NKT function in PBMC stimulated overnight with CD1d/αGC was evaluated in SIV-infected sooty mangabeys, cynomolgus macaques, and rhesus macaques and compared with SIV-negative animals (Table 2). NKT lymphocytes in SIV-infected sooty mangabeys maintained their ability to secrete IFN-γ, IL-2, IL-13, IL-10, IL-6, and TGF-β at levels comparable to, or higher than SIV-negative sooty mangabeys (Fig. 5A and Table 2). In contrast, there was a profound and global decline in NKT functionality in SIV-infected cynomolgus and rhesus macaques (Fig. 5A and Table 2). A greater than 70% reduction in NKT activation-induced secretion of IFN-γ, IL-2, and IL-10 was observed in SIV-infected as compared to SIV-negative macaques (Fig. 5A and Table 2). Significantly, the reduction in cytokine secretion in SIV-infected macaques was largely NKT-specific because, with the exception of IL-6, it was not evident on mitogen stimulation (Fig. 5B).

**Table 2:** Comparison of ex vivo NKT function in SIV-negative and SIV-infected sooty mangabeys (SM), rhesus macaques (RM) and cynomolgus macaques (CM).

	Mean (Range) in pg/ml					
	SM		RM		CM	
Cytokines	SIV−*(n = 6–13)	SIV+(n = 9–14)	SIV−(n = 6–31)	SIV+(n = 6–11)	SIV−(n = 6–17)	SIV+(n = 4–9)
IFN-γ	505	494	172	45†	174	14†
	(143–735)	(119–997)	(13–546)	(0–85)	(45–471)	(0–42)
IL-2	117	324†	95	7†	115	0†
	(5–482)	(89–482)	(0–342)	(0–33)	(0–393)	
IL-13	53	71	5	0	2	0
	(0–155)	(0–211)	(0–43)		(0–11)	
IL-10	87	125	144	0†	75	21†
	(26–140)	(35–218)	(19–252)		(0–155)	(0–54)
IL-6	236	162	51	43	300	191†
	(64–411)	(10–442)	(0–119)	(0–119)	(185–386)	(122–252)
TGF-β	123	87	45	17†	134	57†
	(62–174)	(50–144)	(36–55)	(0–34)	(89–202)	(29–78)

* Range of animal numbers in which assay was performed.

† p<0.05 for SIV− vs SIV+ comparison.

**Figure 6:**
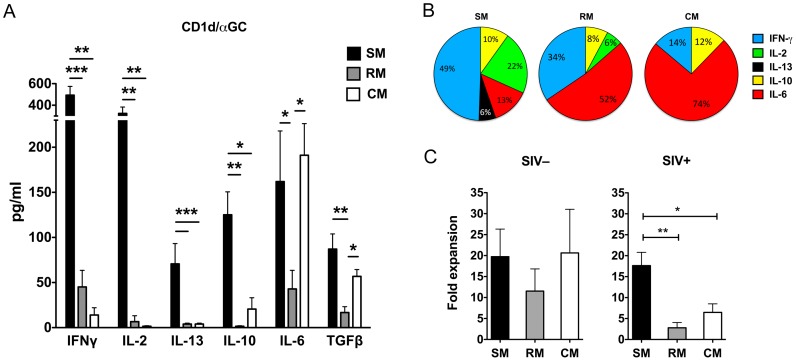
Comparison of NKT lymphocyte function in SIV-infected sooty mangabeys, rhesus macaques and cynomolgus macaques. (A) Amounts of IFN-γ, IL-2, IL-13, IL-10, IL-6, and TGF-β production in culture supernatants collected 24 h following *in vitro* stimulation of SIV-infected SM (n = 9–14), RM (n = 6–11) and CM (n = 4–9) PBMC with CD1d/αGC. Error bars denote SEM. B) Pie graphs showing amounts of individual cytokines as percentage of total amounts of IFN-γ, IL-2, IL-10, IL-13, and IL-6 produced by SIV-infected SM, RM, and CM respectively. C) Proliferation of NKT lymphocytes in PBMC from SIV-negative and SIV-infected SM, RM and CM expressed as fold expansion over a two week period following in vitro stimulation with CD1d/αGC. * *P*<0.05, ** *P*<0.01 Mann-Whitney U test.

Consistent with the disparate effect of SIV infection on NKT function in macaques and mangabeys, NKT lymphocytes from both SIV-infected macaque species showed significantly lower levels of IFN-γ, IL-2, IL-10, and IL-13 secretion in response to CD1d/αGC stimulation when compared to NKT lymphocytes of SIV-infected sooty mangabeys (Fig. 6A). The decline in individual cytokines resulted in profound overall reduction of cytokines produced on NKT activation by SIV-infected macaques (Fig. 6A). Thus, even though the total frequency of circulating NKT lymphocytes remained unchanged in SIV-infected sooty mangabeys and SIV-infected macaques, SIV infection led to significant NKT hypo-functionality in the AIDS-susceptible species.

The residual NKT-dependent function in PBMC of SIV-infected macaques was characterized by a relative dominance of the pro-inflammatory cytokine IL-6 (Fig. 6B). The proportion of IL-6 taken as a percentage of the total amount of IFN-γ, IL-2, IL-10, IL-6, and IL-13 secreted following NKT activation was significantly higher in both SIV-infected rhesus and cynomolgus macaques (mean 52% and 74% respectively) compared to SIV-infected mangabeys (mean 13%; *P*<0.01 Mann Whitney U test) (Fig. 6B).

In addition to impaired cytokine secretion, NKT lymphocytes in SIV-infected macaques also showed a proliferative defect. In the absence of SIV infection, in vitro stimulation of NKT lymphocytes with CD1d/αGC for two weeks resulted in a comparable 11- to 21-fold expansion in sooty mangabeys and macaques (Fig. 6C). However, NKT activation in SIV-infected rhesus and cynomolgus macaques resulted in a significantly lower level of NKT expansion compared to SIV-infected sooty mangabeys (Fig. 6C), indicating that SIV infection also induced NKT hypo-proliferation in macaques.

**Figure 7:**
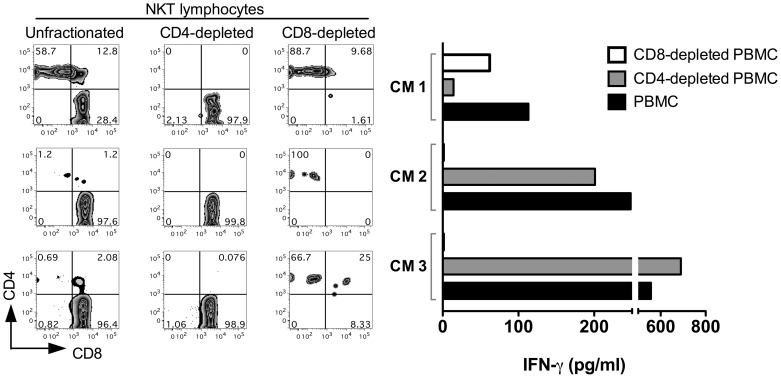
IFN-γ production by cynomolgus macaque NKT lymphocyte subsets. Contour plots showing CD4 and CD8 expression on NKT lymphocytes from 3 SIV-negative CM (CM1, CM2 and CM3). Bar histograms on the right show IFN-γ production by respective cynomolgus macaque PBMC either depleted of CD8^+^ subsets (empty bars) or CD4^+^ subsets (grey bars), in response to 24 h CD1d/αGC stimulation. IFN-γ amounts secreted by NKT lymphocytes in PBMC not depleted of either CD8^+^ or CD4^+^ cells are denoted as black bars. Depletion of CD8^+^ T cells from CM2 and CM3 prior to stimulation with CD1d/αGC resulted in abrogation of IFN-γ production by NKT lymphocytes.

The decreased functionality of NKT lymphocytes in SIV-infected macaques could be due to both loss of the CD4^+^ NKT subset, as well as dysfunction of the surviving CD8^+^ NKT subsets. Data on NKT functionality from SIV-negative cynomolgus macaques showed an equal capability of the CD8^+^ and CD4^+^ NKT subsets to produce IFN-γ and TNF-α (Fig. 2D–E). Moreover, in vitro depletion of CD8^+^ NKT cells prior to stimulation of PBMC with CD1d/αGC resulted in partial to complete abrogation of IFN-γ production (Fig. 7). These data confirm the Th1 functionality of ex vivo CD8^+^ NKT lymphocytes in SIV-negative macaques, and suggest that functional impairment of surviving CD8^+^ NKT lymphocytes is an important factor in the NKT hypo-responsiveness in SIV-infected macaques.

### Loss of CD4^+^ NKT lymphocytes is associated with increased immune activation in SIV-infected macaques

**Figure 8:**
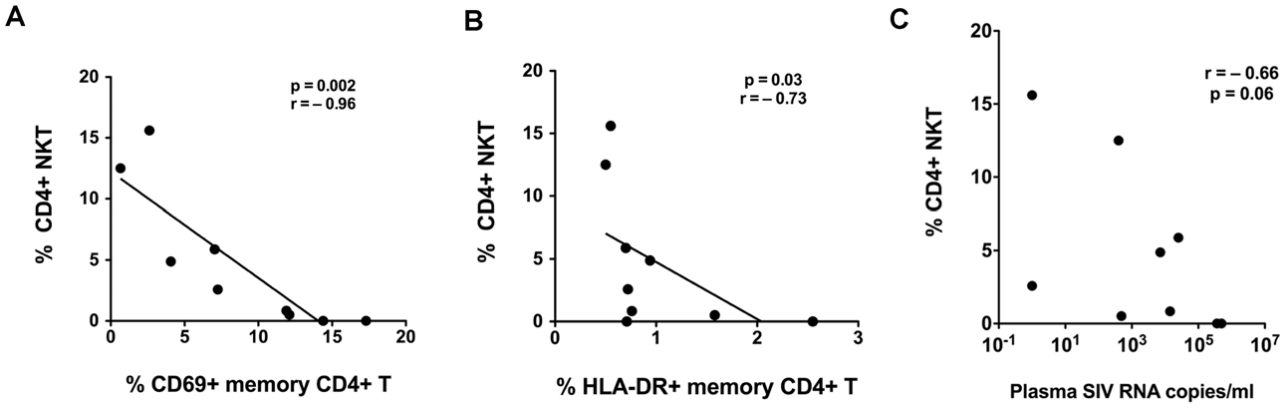
Relationship between CD4^+^ NKT lymphocyte decline and T cell activation and SIV viral loads in SIV-infected cynomolgus macaques. Correlation between proportion of CD4^+^ NKT lymphocytes and expression of the activation markers (A) CD69 or (B) HLA-DR on peripheral blood memory CD4^+^ T lymphocytes (CD4^+^ CD95^+^ T cells). C) Correlation between plasma SIV RNA and frequency of CD4^+^ NKT lymphocytes. *P*-values shown for Spearman rank correlation test. Data in 9 SIV-infected CM shown.

To determine whether SIV-related effects on NKT lymphocytes had an impact on immune activation in chronic SIV infection, we investigated the relationship between NKT lymphocyte frequency and T cell activation levels in nine SIV-infected cynomolgus macaques. The frequency of circulating CD4^+^ NKT lymphocytes showed a strong and significant inverse correlation with the frequency of CD69^+^ and HLA-DR^+^ memory CD4^+^ T lymphocytes suggesting that loss of CD4^+^ NKT was associated with increased immune activation in SIV-infected cynomolgus macaques (Fig. 8A–B). Consistent with previous studies on HIV and SIV infection, we also observed a significant correlation between peripheral CD4^+^ T lymphocytopenia and CD4^+^ NKT depletion (data not shown), and an inverse correlation between the frequency of circulating CD4^+^ NKT lymphocytes and plasma SIV RNA (Fig. 8C).

**Figure 9:**
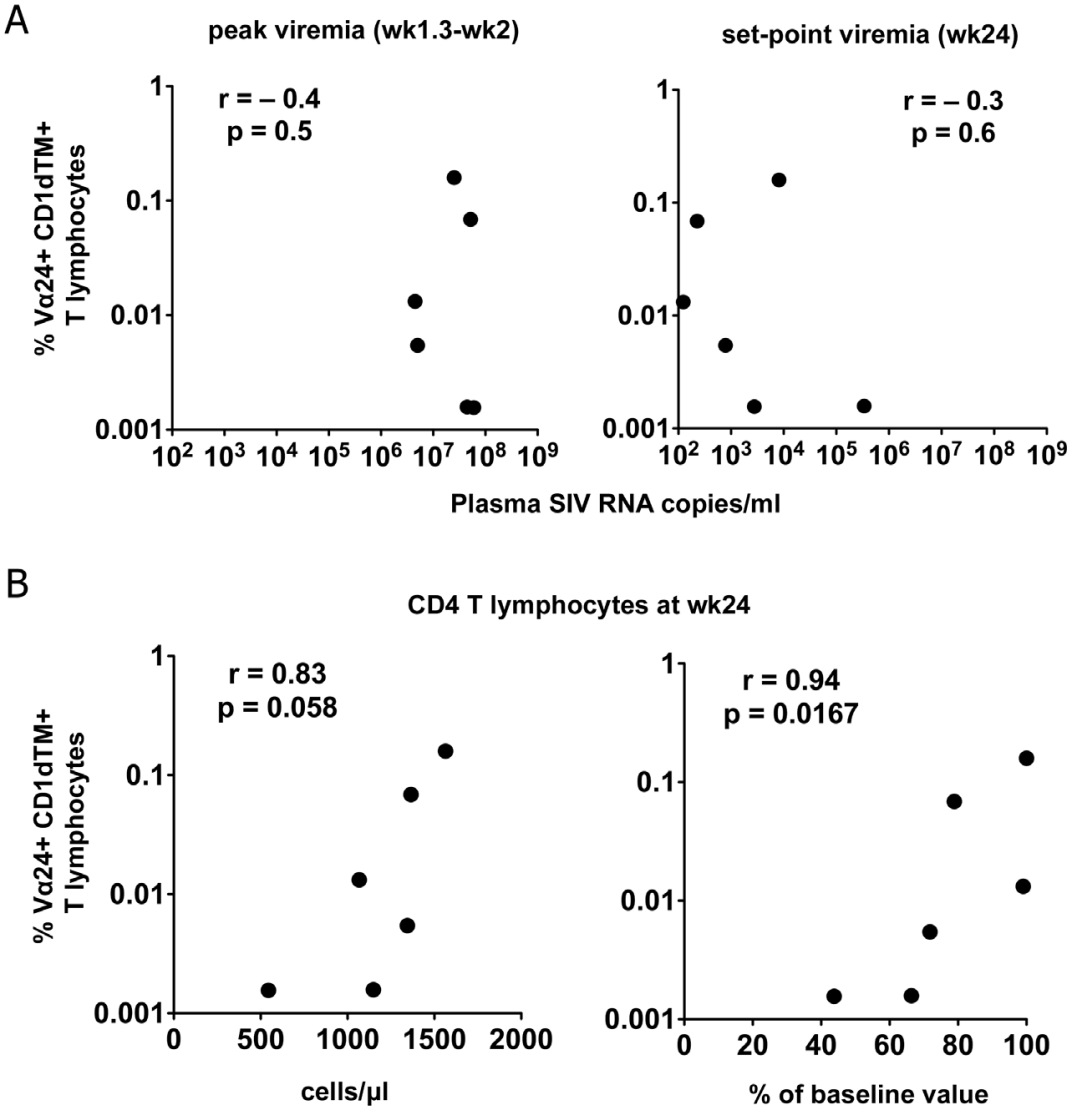
Relationship between pre-infection NKT lymphocyte frequency and outcome of SIV infection in cynomolgus macaques. Correlation between peripheral blood iNKT frequency prior to SIV infection and (A) peak (left panel) and set-point viremia (right panel), and (B) peripheral blood CD4^+^ T lymphocyte counts (left panel) and CD4^+^ T cell loss (right panel) post SIV infection in six cynomolgus macaques. Data at 10–14 days post SIV infection for peak viremia and at week 24 post SIV infection for set-point viremia and CD4^+^ T lymphocyte counts shown. CD4^+^ T lymphocyte loss at week 24 was expressed as a percentage of pre-SIV infection values. *P*-values and r-values calculated by the Spearman rank correlation test.

Because of the cross-sectional nature of this study, it is not possible to determine whether the association between CD4^+^ NKT loss and increased immune activation indicated a causal relationship, or reflected the effect of advanced immunodeficiency in chronic SIV infection. To explore the possibility that NKT lymphocytes can modulate progression to AIDS, we investigated whether NKT frequency prior to SIV infection can affect viral load or CD4^+^ T lymphocyte loss post SIV infection. Limited availability of archived cryopreserved PBMC allowed determination of pre-infection peripheral blood NKT lymphocyte frequencies in six of the nine SIV-infected cynomolgus macaques (Fig. 9). Although pre-infection NKT frequencies did not correlate with peak or set-point viremia (Fig. 9A), there was a positive correlation with CD4^+^ T cell counts at 24 weeks post SIV infection, either expressed as absolute counts or as percent of baseline levels (Fig. 9B). These findings, albeit in a small number of animals, raise the possibility that pre-infection NKT frequencies are a determinant of the rate of CD4^+^ T cell loss and disease progression in chronic SIV infection.

## Discussion

We previously reported on the unique lack of CD4^+^ NKT lymphocytes in sooty mangabeys, a phenotype that likely protects them from NKT depletion following SIV infection [23]. This contrasts with several reports of NKT depletion in HIV-infected humans [16], [17], [18], [27], [28], and more recently in SIV-infected pigtail macaques [19]. However, the consequences of NKT depletion on outcome of HIV/SIV infection remain unclear. Because NKT lymphocytes are rapid responders of the innate immune system and have potent immunoregulatory properties, we hypothesized that differences in NKT activation or function may contribute to the differential modulation of immune activation in pathogenic and nonpathogenic SIV infection in non-natural and natural hosts respectively. NKT lymphocytes also interact with several immune cell subsets including DCs, NK cells, CD4^+^ T helper cells and B lymphocytes, and function as an important bridge between innate and adaptive immunity [29]. Thus, loss of NKT function may also be involved in the pathogenesis of HIV/SIV-induced immunodeficiency [30], [31]. In this study we compared two AIDS-susceptible species of Asian macaques, Mauritian cynomolgus macaques and Indian rhesus macaques, with the AIDS-resistant natural host sooty mangabeys. We detected significant phenotypic and functional differences in ex vivo NKT lymphocytes between sooty mangabeys and the two macaque species. These differences were more pronounced in the setting of chronic SIV infection. The impaired NKT activation in SIV-infected macaques encompassed several functions; loss of IFN-γ and IL-2 compromising NKT effector function, loss of IL-10 and TGF-β leading to decreased anti-inflammatory function, and the relative sparing of IL-6 production, contributing to overall increased inflammation. Although the cross-sectional nature of this study does not allow for definitive conclusions on causality, in this first comparative study of NKT lymphocytes in natural and non-natural hosts of SIV, our findings suggest that NKT dysfunction has a role in AIDS pathogenesis.

The frequency of circulating invariant NKT lymphocytes was not predictive of the magnitude of NKT-dependent functional responses. Despite cynomolgus macaques having a 10- to 100-fold higher NKT frequency compared to rhesus macaques and sooty mangabeys, both macaque species shared several functional similarities that differentiated them from sooty mangabey NKT lymphocytes. Compared to mangabeys, ex vivo NKT lymphocytes from macaques secreted less IFN-γ, and little or no IL-13 on activation with αGalCer presented on CD1d-expressing transfectant cell lines. Neither the mangabeys nor the macaques showed detectable IL-17 release from stimulated ex vivo NKT lymphocytes. However, IL-17 was detected from in vitro cultured NKT cell lines of sooty mangabeys, but not macaques. Rather than an intrinsic qualitative difference in NKT function between species, it appears more likely that the differences in IL-13 and IL-17 secretion are of a quantitative nature and may reflect functional differences in peripheral blood NKT lymphocytes in the respective species. Thus, robust IL-13 production has been reported from cultured splenic-derived NKT cell lines in rhesus macaques [32]. Similarly, increased IL-17-secreting NKT cells were detected in the lymph node paracortex of SIV-infected rhesus macaques and were associated with a poorer disease outcome [33]. Overall, our data suggest that ex vivo peripheral blood NKT responses are significantly more potent in sooty mangabeys compared to macaques. If this trend holds true for the in vivo NKT response to pathogens, it could have profound implications for the nature of the early host response to SIV infection in the respective species.

Could the phenotypic differences account for the differences in NKT functionality between the species? The presence or lack of CD4 expression can delineate functionally distinct subsets of NKT lymphocytes in mice and humans [24], [25]. Secretion of immunoregulatory Th2 cytokines and IL-10 are generally associated with the CD4^+^ subset of human and nonhuman primate NKT lymphocytes, whereas DN NKT tend to have more effector properties in terms of Th1 cytokine secretion, cytotoxicity and greater anti-tumor efficacy [34]. The lack of CD4^+^ NKT lymphocytes does not appear to be a functional barrier in sooty mangabeys because as we previously reported, the DN NKT cells of mangabeys are functionally CD4-like with regards to Th2 cytokine secretion [26]. Furthermore, both CD8^+^ and DN NKT also secrete IL-2 and IFN-γ in sooty mangabeys. Since CD4^+^ NKT cells are highly susceptible to lentiviral infection [16], [35], sooty mangabeys may have evolved to avert CD4 expression on their NKT cells to avoid being targeted and depleted by direct SIV infection without compromising NKT function. We did not detect any impairment of NKT function in the naturally SIV-infected sooty mangabeys investigated in this study. On the contrary, IL-2 production was significantly higher in NKT lymphocytes from SIV-infected sooty mangabeys in comparison to SIV-negative animals. IL-2 administration in HIV-infected patients can result in substantial expansion of conventional CD4^+^ T cells [36] including naïve, memory and regulatory T cell subsets [37] as well as NKT and NK cells [38], [39]. Besides, IL-2 and IFN-γ production by human NKT cells has been shown to strongly activate NK cell cytotoxicity against tumor cell lines [40]. Also, human NKT cells have demonstrated potent suppression of HIV-1 replication via IFN-γ secretion [41]. Thus, preserved IFN-γ and other cytokine responses combined with an enhanced IL-2 production might be important in maintaining immune homeostasis in SIV-infected sooty mangabeys.

In addition to depletion of CD4^+^ NKT subsets, SIV-infected macaques also showed a marked hypo-functionality of surviving CD8^+^ NKT lymphocytes. Several studies have reported functional impairment of NKT lymphocytes in HIV-infected patients [27], [28], [38], [39], [41], [42]. The mechanisms of functional impairment remain to be elucidated; PD-1 can be upregulated on NKT cells in HIV-infected individuals but PD-1 blockade did not improve in vitro function [27]. HIV has also developed strategies to evade NKT cell activation by downregulating CD1d cell surface expression on antigen presenting cells [20], [21], [22]. HIV Nef enhances CD1d endocytosis, while Vpu retains CD1d in the ER [20], [22]. Thus, impaired NKT immunity in HIV-infected individuals may be a result of both functional anergy of NKT lymphocytes as well as a defect of antigen presentation and in vivo NKT activation.

In conjunction with the hyporesponsiveness, the residual function of NKT lymphocytes in SIV-infected macaques was dominated by IL-6 induction. Although the functional impact of this observation is not known, it is possible that a skewing of NKT function towards inducing the proinflammatory cytokine IL-6 combined with a decline in IL-10 and Th2 cytokine production could result in overall increased inflammation [43]. It was noteworthy that CD4^+^ NKT decline correlated with increased CD4^+^ memory T cell activation, suggesting that NKT depletion may contribute to increased immune activation in SIV-infected macaques. With the caveat that cross-sectional analysis precludes definitive conclusions regarding a direct causal link between NKT dysfunction and increased immune activation from this study, there are several possible mechanisms by which NKT cells could modulate immune activation in HIV infection. NKT cells can have a direct suppressive effect via secretion of anti-inflammatory cytokines such as IL-10, IL-4, and IL-13. In this regards, the discrepancy in IL-13 secretion by ex vivo NKT cells between macaques and mangabeys is interesting. IL-13 was originally described as a T cell–derived cytokine that inhibits inflammatory cytokine production [44], [45]. Although studies have implicated IL-13 in the induction of allergy or asthma, and hepatic fibrosis [46], [47], it has also been shown to suppress inflammation in the setting of proinflammatory immune activation, either by inducing activation of macrophages with anti-inflammatory properties [48], [49] or by inducing TGF-β production from immature myeloid cells [50], [51]. There is also evidence that NKT cells are activated by myeloid DCs in a negative feedback loop such that excessive Th1 cytokines by DCs induces Th2 secretion in NKT and thus dampens inflammation [52], [53]. NKT cells can also modulate immunoregulation by its effect on other cell populations. Thus, NKT cells can drive Tregs towards increased IL-10 production via upregulation of PD-1 [54]. IL-6 and TGF-beta induced by NKT may promote Th17 differentiation. Additionally there are IL-17-producing NKT that express IL23r and RORγT and secrete IL-17 in an IL-6-independent fashion [55]. Preservation of Th17 cells has been associated with intact mucosal integrity in natural hosts of SIV infection [56]. Whether other IL-17-secreting populations such as NKT cells would play a similar role remains unknown.

In the absence of longitudinal data, a causal link between NKT dysfunction and AIDS pathogenesis cannot be definitively established. It is conceivable that NKT dysfunction in the SIV-infected macaques and intact NKT function in the SIV-infected mangabeys were a result of the overall disease stage in the respective AIDS-susceptible and AIDS-resistant species. While we cannot exclude this possibility, it is noteworthy that the SIV-infected macaques showed profound functional impairment of NKT activation-induced responses but not PMA-induced responses (Fig. 5) suggesting that there was disproportionate NKT hypo-function without overt global immunodeficiency. It is also interesting that in a small subset of macaques with available cryopreserved samples, we detected a significant association between pre-infection NKT frequencies and preservation of CD4^+^ T cells post SIV infection. If confirmed in prospective studies, these data suggest a protective role of NKT cells in slowing down the rate of CD4^+^ T cell decline. Future longitudinal and interventional studies in the presence and absence of NKT depletion are required to interrogate the causal role of NKT cells in downmodulating immune activation and preventing immunodeficiency in AIDS.

In this study, we demonstrate for the first time differences in NKT function between sooty mangabeys and two Asian macaque species. Our results suggest that loss of anti-inflammatory and effector NKT function in SIV-infected macaques may have a role in AIDS pathogenesis, whereas intact NKT function may protect natural hosts from immunodeficiency and increased immune activation. Future interventional studies with in vivo NKT activation or NKT depletion experiments in nonhuman primates will be invaluable to dissect the role of NKT lymphocytes in protection against AIDS.

## Materials and Methods

### Ethics statement

This study was carried out in strict accordance with the recommendations in the Guide for the Care and Use of Laboratory Animals of the National Institutes of Health. The animal protocol was approved by the Harvard Medical School Area Standing Committee on Animals. This institution has an approved Animal Welfare Assurance on file with the Office for Laboratory Animal Welfare (Assurance number A3431-01).

### Animals

Sooty mangabey blood samples used in this study were obtained from SIV-negative and naturally SIV-infected animals housed at the Yerkes National Primate Research Center (YNPRC), Atlanta. The SIV-negative Indian rhesus macaques, SIV-negative Mauritian cynomolgus macaques and SIVmac239-infected rhesus macaques were housed at the New England Primate Research Center (NEPRC), MA. SIVmac239-infected Mauritian cynomolgus macaque blood samples were obtained from animals housed at the Wisconsin National Primate Research Center (WNPRC), WI. All animals were maintained in accordance with institutional and federal guidelines for animal care (National Research Council. 1996).

### Preparation of peripheral blood mononuclear cells

Sooty mangabey blood was collected at YNPRC in heparin vacutainer tubes (Becton Dickinson Vacutainer systems, Franklin Lakes, NJ), and shipped overnight on wet ice to NEPRC where it was processed the following day. SIVmac239-infected cynomolgus macaque blood was similarly collected at WNPRC and shipped to be processed on the following day. Peripheral blood mononuclear cells (PBMC) were separated by density gradient centrifugation (Lymphocyte Separation Medium; MP Biomedicals Inc., Solon, OH) at 1500 rpm for 45 minutes and used for phenotyping and in vitro assays.

### Immunophenotyping and flow cytometry of NKT cells

Multi-color flow cytometric analysis was performed on cells according to standard procedures using anti-human mAbs that cross-react with rhesus macaques. NKT lymphocyte ligand PBS-57-loaded and unloaded human CD1d Tetramers (CD1d TM) conjugated with APC were obtained from the NIH Tetramer core facility. The following antibodies were obtained from BD Biosciences unless stated otherwise: anti-Vα24–PE (clone C15; Immunotech), anti-CD3–APC-Cy7 (SP34-2), anti-CD4–Qdot605 (T4/19Thy5D7; custom/NHP Resource), anti-CD8–Alexa Fluor 700 (RPA-T8), anti-IFN-γ–PE-Cy7 (B27), anti-IL-2–APC (MQ1-17H12), anti-IL-13–FITC (PVM13-1; eBioscience), and anti-TNF-α–Alexa Fluor 700 (MAb11).

For identification of NKT cells, PBMC were surface stained for CD3 and anti-Vα24 combined with PBS-57 loaded CD1d TM. APC-labeled unloaded CD1d TM controls were used in all experiments. Surface staining was carried out by standard procedures. Briefly, 2 to 4 million PBMC resuspended in 100 µl wash buffer (PBS with 2% FBS) were initially incubated with tetramers and Vα24 antibody for 20 min at 4°C followed by addition of surface antibodies and further incubation for 30 min at 4°C. After washing, the cells were fixed in 2% paraformaldehyde. All intracellular cytokine staining (ICS) assays were carried out on cells that were stimulated overnight. Following 16 h incubation, cells were washed in PBS containing 2% FCS and 0.5 mM EDTA, stained for surface markers in wash buffer for 30 min at 4°C, washed and then fixed and permeabilized using the Invitrogen Fix/Perm reagents (CALTAG™). Permeabilized cells were stained intracellularly with the requisite antibodies. Cells were then washed in wash buffer and fixed in 2% paraformaldehyde. Flow cytometric acquisition was performed on an LSR-II cytometer driven by the FACS DiVa software (version 5.2; BD). At least 200,000 T lymphocyte events were collected. Analysis of the acquired data was performed using FlowJo software (version 8.8.3; TreeStar, Ashland, OR).

### Medium and reagents

The complete medium (R10 medium) used throughout was RPMI medium 1640 (Cellgro, Herndon, VA) supplemented with 10% FCS (Sigma-Aldrich, St. Louis, MO), 1% 1 M HEPES, 2 mM L-glutamine (Cellgro), 50 IU/ml penicillin (Cellgro), 50 µg/ml streptomycin (Cellgro). The NKT-ligand α-galactosylceramide (α-GalCer) and anti-human CD1d antibody (Clone 42.1) were a gift from Mark Exley (BIDMC, HMS, Boston) and were used at 100 ng/ml and 0.1–20 µg/ml respectively. Recombinant human IL-2 (Roche) was used at 10–50 IU/ml of medium for the in vitro expansion of NKT cells.

### In vitro expansion of NKT cells

Freshly isolated PBMCs (10^6^ cells) from individual SIV-negative and SIV-infected animals were incubated in R10 medium containing 100 ng/ml of α-GalCer along with 100,000 cells of CD1d-transfected C1R B cell line (C1R.d) irradiated at 3000 rads. After two days, 50 IU/ml recombinant human IL-2 supplemented medium was added to the cultures and stimulated cells were expanded for 2 weeks. Functional evaluation for cytokine producing ability was done by restimulating 500,000 cells with 100 ng/ml of α-GalCer for 16 hours followed by ICS assay as earlier described. The proliferation of NKT cells was confirmed by staining with anti-Vα24 and PBS-57 loaded CD1dTM. Expansion of NKT lymphocytes was measured as fold increase in frequencies of Vα24^+^CD1d TM^+^ CD3^+^ T cells from initial ex vivo frequencies detected in individual animals.

### Functional analysis of NKT lymphocytes

PBMC from individual SIV-negative and SIV-infected animals were simultaneously thawed and cultured in triplicate, using 10^5^ cells/well in a 96-well flat-bottom plate. Cells were stimulated with medium alone, 25 ng/ml PMA (Sigma-Aldrich, St. Louis, MO) with 1 µg/ml Calcium (PMA/Ca), or with APCs that had been pulsed with α-GalCer at a final concentration of 100 ng/ml. 50,000 C1R.d cells were γ-irradiated at 10,000 rads and used as APCs for the presentation of α-GalCer as previously described [57]. Irradiated mock-transfected C1R cells served as a negative control stimulus for NKT cells. After 24 h, culture supernatants were collected and stored frozen at −20°C. Inteferon (IFN)–γ, IL-2, IL-6, IL-10, IL-13, IL-17, and TGF-β were detected in supernatants from all animals (SIV-negative and SIV-infected) by use of ELISA for monkey cytokines (U-CyTech, Utrecht, Netherlands). Cytokine ELISAs (U-CyTech BV Diagnostics) were performed according to the manufacturer's instructions. Levels of cytokines (pg/mL) were interpolated from standard curves. Data are expressed as mean ± SEM cytokine production from each group of 5–10 animals.

### Statistical analysis

Statistical differences between groups were determined by use of the Mann Whitney U test. The Pearson test was performed for correlation analysis. P<0.05 was considered statistically significant. All statistical analyses were performed using the GraphPad Prism software version 5.0b (GraphPad Software, Inc., La Jolla, CA). All data are presented as mean ± SEM.
